# Senescence-Associated Molecular and Epigenetic Alterations in Mesenchymal Stem Cell Cultures from Amniotic Fluid of Normal and Fetus-Affected Pregnancy

**DOI:** 10.1155/2016/2019498

**Published:** 2016-10-10

**Authors:** Jūratė Savickienė, Sandra Baronaitė, Aistė Zentelytė, Gražina Treigytė, Rūta Navakauskienė

**Affiliations:** Department of Molecular Cell Biology, Institute of Biochemistry, Vilnius University, LT-10257 Vilnius, Lithuania

## Abstract

Human amniotic-fluid-derived mesenchymal stem cells (AF-MSCs) are interesting for their multilineage differentiation potential and wide range of therapeutic applications due to the ease of culture expansion. However, MSCs undergo replicative senescence. So far, the molecular mechanisms that underlie fetal diseases and cell senescence are still poorly understood. Here, we analyzed senescence-associated morphologic, molecular, and epigenetic characteristics during propagation of MSCs derived from AF of normal and fetus-affected pregnancy. AF-MSCs cultures from both cell sources displayed quite similar morphology and expression of specific cell surface (CD44, CD90, and CD105) and stemness (Oct4, Nanog, Sox2, and Rex1) markers but had interindividual variability in proliferation capability and time to reach senescence. Within passages 4 and 8, senescent cultures exhibited typical morphological features, senescence-associated *β*-galactosidase activity, increased levels of p16, and decreased levels of miR-17 and miR-21 but showed differential expression of p21, p53, and ATM dependently on the onset of cell senescence. These differences correlated with changes in the level of chromatin modifiers (DNMT1 and HDAC1) and polycomb group proteins (EZH2, SUZ12, and BMI1) paralleling with changes in the expression of repressive histone marks (H3K9me3 and H3K27me3) and stemness markers (Oct4, Nanog, Sox2, and Rex1). Therefore epigenetic factors are important for AF-MSCs senescence process that may be related with individuality of donor or a fetus malignancy status.

## 1. Introduction

Human amniotic fluid (AF) contains multiple cell types, including about 1% of cells with mesenchymal stem cell characteristics [1]. These amniotic-fluid-derived stem cells (AF-MSCs) can be obtained from a small amount of AF of second trimester pregnancy through procedure of amniocentesis used for prenatal diagnosis to determine the fetus status and genetic diseases [2, 3]. AF possesses several advantages such as safe and easy sampling, little maternal and child trauma, and no ethical restrictions. To date, AF is considered as an attractive source of MSCs having great potential application in regenerative medicine because of their extensive self-renewal, multilineage differentiation capacities [4–6], low or negligible immunogenicity, and inability to form tumors after implantation [7, 8]. However, the extended stem cell cultivation through sequential passaging* in vitro* may lead to a state of replicative senescence. It has been suggested that human MSCs derived from different sources become senescent as indicated by their morphological changes, attenuated expression of specific surface markers, decreased proliferation and differentiation potential, telomere length shortening, and alterations in global gene expression, DNA methylation patterns, and miRNA profiles [9–11]. Clearly, cellular senescence is a complex process and molecular events associated with AF-MSCs* in vitro* expansion are thus far unknown.

Primary somatic cells can replicate in culture about 50 cumulative population doublings, after which the cultures stop dividing. This phenomenon is termed Hayflick's limit and is known as replicative senescence [12]. MSCs have a limited lifespan* in vitro* as any normal, somatic cells and are also subject to the Hayflick limit. It was suggested that replicative senescence of MSCs is a continuous process starting from the first passage [11], leading to seriously affected proliferative and clonogenic potential [11, 13] and related to morphological and molecular characteristics. Proliferation arrest within 10–20 passages was observed in senescent MSCs undergoing morphological and phenotype alterations [11, 14, 15] together with changes in the global gene expression pattern at different passages and significant downregulation of genes involved in cell cycle, DNA replication, and DNA repair in late passages [11]. By the use of gel-based proteomic method [16], the variety of fluctuations was observed for chaperones, signaling, antioxidant, proteasome, cytoskeleton, and connective tissue proteins in human AF stem cell line CD117/2 culture along passages 5–7 and 25. Two-dimensional gel electrophoresis-based quantitative proteomics in bone marrow MSCs cultures at passages 3–7 demonstrated also differential protein expression profiles associated with the impairment of cytoskeleton remodeling and/or organization and the repair of damaged proteins [17]. There is also evidence that senescence involves DNA damage, oxidative stress, a decrease in multilineage differentiation potential, and telomerase activity [15, 18, 19].

Several biomarkers have been used for quantitative assessment of cell senescence, including senescence-associated *β*-galactosidase (SA-*β* gal) and the cyclin-dependent kinase inhibitors, p16INK4A and p21WAF1, involved in the control of growth arrest by two major tumor suppressor pathways, p16INK4A/pRb and p53/p21WAF1 [20, 21]. A potential marker of the senescent state of stem cells may serve decreased expression of pluripotency transcription factors, such as Nanog and Oct4 [22, 23].

Replicative senescence seems to be epigenetically controlled [24]. Recent studies indicate that senescence-associated DNA methylation changes are associated with repressive histone marks and with targets of the histone methyltransferase EZH2, a component of polycomb complex PRC2 [25–27]. However, epigenetic regulation mechanisms underlying morphologic and phenotypic changes in MSCs during culture expansion are still unclear.

It is known that there exists different proliferative capability of MSCs cultures isolated from different donors [11, 13], undergoing senescence and considerable property changes. In order to further explore the observation about growth disadvantages, the present study aimed to examine how donor individuality and fetus abnormalities may influence amniotic-fluid-derived MSCs state during culture expansion. Here we provide evidence that MSCs cultures from AF of normal gestation and with fetus abnormalities exhibited diversity in their proliferation and senescence. We describe senescence-associated molecular and epigenetic changes during MSCs cultivation related with the extent of MSCs characteristics.

## 2. Material and Methods

### 2.1. Amniotic-Fluid-Derived MSCs Isolation, Culture, and Expansion

AF samples (about five milliliters) were obtained by biopsy (amniocentesis) from mid-second-trimester (16–24 weeks) or third-trimester (28–34 weeks) pregnant woman who needed prenatal diagnosis, and no abnormalities were revealed by genetic analysis using protocols approved by the Ethics Committee of Biomedical Researches of Vilnius District, number 158200-123-428-122. Samples were maintained at room temperature for about 4 hours prior to isolation of amniotic cells using two-stage protocol [23]. The sample was centrifuged at 1,800 rpm for 20 min, the supernatant was removed, and the cell pellet was washed once in DMEM medium (Sigma-Aldrich Ltd.) without serum to remove blood and cell debris. After centrifugation, the cell pellet was resuspended in 5 mL of growth medium (AmnioMAX*™*-C100 basal medium with AmnioMAX*™*-C100 supplement (Gibco, Life Technologies, Grand Island, NY, USA)), containing 100 U/mL penicillin, and 100 *μ*g/mL streptomycin (Gibco, Grand Island, NY, USA) and plated in a 25 cm^2^ culture flask (TPP, Switzerland). Amniocytes were incubated for 10–15 days at 37°C in 5% CO_2_, when first colonies appeared (first stage). For culturing MSC (second stage), nonadhering AF cells were collected from primary culture and further expanded in a new 25 cm^2^ culture flask at 37°C in 5% CO_2_. After the appearance of the cell colonies, the growing medium was changed every 3 days. When cells reached confluence at 80%, subculturing into higher passages was performed by trypsinization with 0.05% trypsin-EDTA (Gibco, Life Technologies, Grand Island, NY, USA) for 3 min. A morphologically homogeneous population of fibroblast-like cells was obtained after two rounds of subculture. Cell morphology was observed by phase-contrast microscope (Nicon Eclipse TS100). Cell population doubling levels are presented as the average values calculated as follows: cell culture time (days)/number of passages.

### 2.2. Flow Cytometry Analysis

For identification of the phenotype of AF-MSCs from passages 4-5, cells were collected by centrifugation at 1,200 rpm for 6 min, washed once in phosphate buffered saline (PBS) with 0.2% fetal calf serum (FCS), and centrifuged again. A total of 5 × 10^5^ cells were resuspended in 50 *μ*L of PBS with 1% BSA and incubated with fluorescein isothiocyanate- (FITC-) conjugated mouse anti-human antibodies against CD44 (Invitrogen), CD34 (Miltenyi Biotech), CD90 (Molecular Probes, Life Technologies), or phycoerythrin- (PE-) labeled CD105 (Invitrogen) and appropriate isotype control—mouse IgG2A-FITC (Miltenyi Biotech) or IgG1-PE (Molecular Probes, Life Technologies). Samples were incubated in the dark at 4°C for 30 min and finally analyzed with the Millipore Guava® easyCyte 8HT flow cytometer, using the InCyte 2.2.2 software. Ten thousand events were collected for each sample.

### 2.3. Senescence-Associated *β*-Galactosidase Assay

Cellular senescence was assessed using the *β*-galactosidase staining kit (Cell Signaling) following the manufacturer's instruction. Cells were seeded in 48-well plates and cultivated for 48 hours. After washing with PBS, cells were fixed with 4% formaldehyde in PBS for 15 min and stained for *β*-galactosidase activity using staining solution overnight at 37°C in a thermostat without CO_2_. Stained cells were viewed under a phase-contrast microscope (Nicon Eclipse TS100) and the data was expressed as the percentage of *β*-galactosidase positive cells.

### 2.4. RNA Isolation and RT-qPCR

MSCs from AF samples of normal gestation and with fetus abnormalities were cultured for 3–8 passages and analyzed for stem cell specific markers. Total RNA was extracted by TRIzol (Applied Biosystems, USA), as recommended by the manufacturer, and then reverse-transcribed into cDNA using Maxima First Strand cDNA Synthesis Kit (Thermo Scientific). qPCR was performed with Maxima® SYBR Green qPCR Master Mix (Thermo Scientific) on the Rotor-Gene 6000 system (Corbett Life Science). The amount of mRNA was normalized to GAPDH. The relative gene expression was calculated by a comparative threshold cycle delta-delta Ct method. Statistical analysis was performed using Student's *t*-test.

Forward (F) and reverse (R) primers (5′-3′) used in RT-qPCR are as follows:

 OCT4: F: CGAGAAGGATGTGGTCCGAG; R: CAGAGGAAAGGACACTGGTC
 Nanog: F: AGATGCCTCACACGGAGACT; R: GTTTGCCTTTGGGACTGGTG
 Sox2: F: TGGACAGTTACGCGCACAT; R: CGAGTAGGACATGCTGTAGGT
 Rex1: F: GCCTTATGTGATGGCTATGTGT; R: ACCCCTTATGACGCATTCTATGT
 p16: F: GCTGCCCAACGCACCGAATA; R: ACCACCAGCGTGTCCAGGAA
 p21: F: GGCAGACCAGCATGACAGATT; R: GCGGATTAGGGCTTCCTCT
 p53: F: TAACAGTTCCTGCATGGGCGGC; R: AGGACAGGCACAAACACGCACC
 ATM: F: CTCTGAGTGGCAGCTGGAAGA; R: TTTAGGCTGGGATTGTTCGCT
 GAPDH: F: TCCATGACAACTTTGGTATCG; R: TGTAGCCAAATTCGTTGTCA

The amount of mRNA was normalized to GAPDH. Normalization and fold changes were calculated using a comparative threshold cycle delta-delta Ct method. Student's *t* test was used for statistical analysis. All values are expressed as mean ± SD. ^*∗*^ *P* value ≤ 0.05 was considered significant.

### 2.5. MicroRNA Expression

The expression of target miRNAs in AF-MSC samples was evaluated by RT-qPCR analysis using probe-based TaqMan MicroRNA Assays. Reverse transcription was performed using TaqMan MicroRNA Reverse Transcription Kit (Applied Biosystems, USA); amplification was performed using TaqMan Universal PCR Master Mix (Applied Biosystems, USA) according to the manufacturer's recommendations. Reactions were conducted in Finnzymes Thermocycler with a program as follows: 16°C for 30 min, 42°C for 30 min, and 85°C for 5 min. The product cDNA of each reaction was used as template for qPCR. RT-qPCR was carried out in a Rotor-Gene 6000 system (Corbett Life Science). The reaction was performed at 95°C for 10 min, followed by 40 cycles of 95°C for 15 s and 60°C for 60 s. RNU 48 gene was used as a reference gene to normalize all experimental data. All reactions were performed in triplicate. The threshold cycle (Ct) was determined using the default threshold settings, and relative quantification of miRNA was calculated with the 2^−ΔΔCt^ method. Student's *t*-test was used for statistical analysis. All values are expressed as mean ± SD. ^*∗*^ *P* values ≤ 0.05 were considered significant.

### 2.6. Preparation of Proteins and Western Blotting

AF-MSCs (about 5 × 10^5^) were harvested by centrifugation (500 ×g, 6 min) after trypsinization with 0.05% trypsin-EDTA, washed twice in ice cold PBS, and resuspended in 10 volumes of lysis solution (62.5 mM Tris, pH 6.8, 100 mM DTT and 2% SDS, 10% glycerol). Benzonase (Pure Grade, Merck) was added to give a final concentration of 2.5 units/mL. Cell lysate was prepared by homogenization through the needle Nr 21 on ice and then centrifuged at 20,000 ×g for 10 min, 4°C. The supernatants were immediately subjected to electrophoresis or frozen at −76°C. Protein concentrations were measured using commercial RCDC protein assay (Bio Rad). The lysates were separated on a 7–15% polyacrylamide gradient SDS-PAGE gel and then transferred to a PVDF membrane. The filters were incubated with the primary antibody according to the manufacturer's recommendations and then with horseradish peroxidase-conjugated (HPR) secondary antibody (Dako Cytomation, Glostrup) at room temperature for 1 h. The bands were developed using enhanced chemiluminescence detection (Amersham Pharmacia) according to manufacturer's instruction. For Western blotting, the following antibodies were used: DNMT1 and EZH2 were from Cell Signaling; H3K9me3 was from Upstate; H3K27me3 and BMI1 were from Millipore; ATM, ATM (phospho S1981), and GAPDH were from Abcam; goat anti-rabbit or rabbit anti-goat HPR (horseradish peroxidase) linked secondary antibodies were from Dako Cytomation A/S.

## 3. Results

### 3.1. Characterization of MSCs Cultures from AF of Normal and Fetus-Affected Pregnancy during Passaging

**Figure 1:**
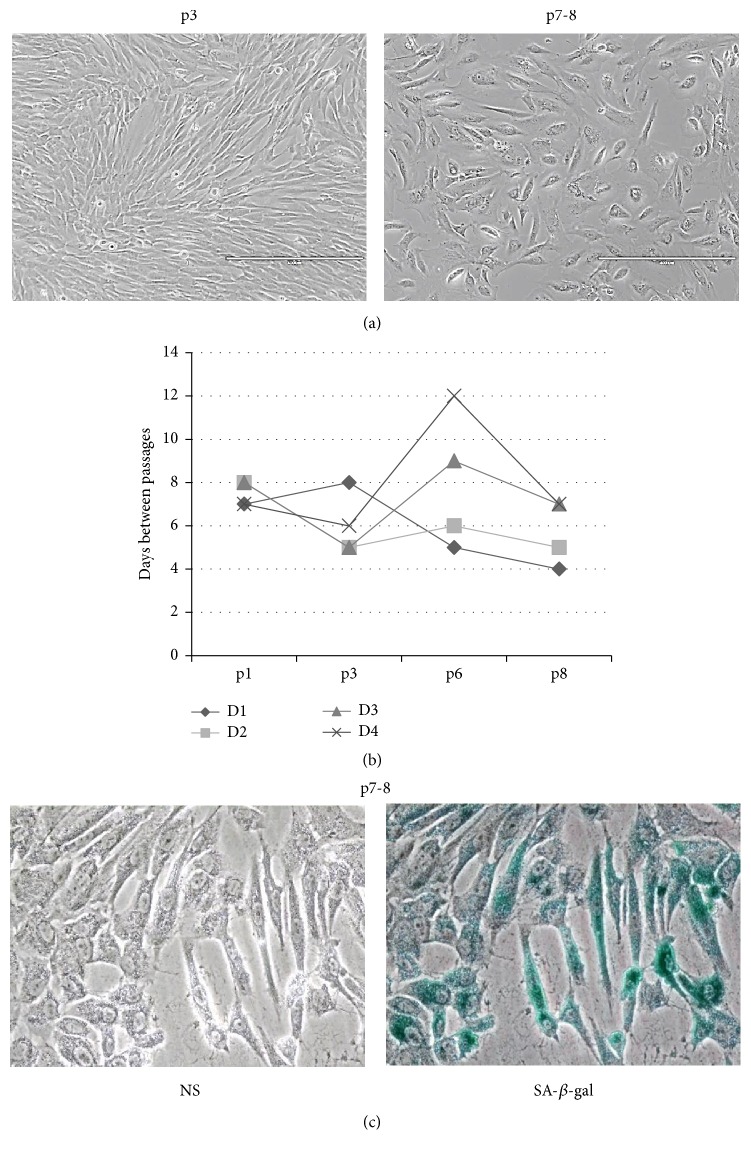
Characteristics of MSCs derived from AF of normal and pathological gestation during cultivation. (a) Representative images of initially spindle-shaped MSCs morphology in early (p3) and senescent passages (p7-8) with a 40x magnification are presented. (b) Differential proliferation of MSCs from AF of individual donors (D) during cultivation to passage 8. (c) Senescence-associated *β*-galactosidase staining in the late passage (p7-8); NS: nonstained cells. Representative results of four independent MSC preparations are demonstrated.

MSCs were independently isolated from AF of healthy (N) donors (D1) and those carrying fetus abnormalities (P), such as D2, fetal central nervous system pathology and dilated brain ventricles; D3, circulatory disorders and increased heart; D4, trisomy 21 (Down's syndrome). MSCs were cultured for 3–9 consecutive passages using AmnioMAX*™*-C100 complete medium. At each passage, MSCs were replated at 80–90% confluence. At early passages (p3-4), cell cultures from both AF sources formed and maintained homogeneous populations of typical elongated mesenchymal-type and spindle-shaped morphology (Figure 1(a)). The proliferation of MSCs achieved maximum on passage 3 (Figure 1(b)). After passages 5-6, there was a decline in the efficiency of proliferation in all cell populations. Time to reach senescence differed between cultures. As shown in Figure 1(b), some donors samples, including AF of normal and fetus-pathological gestation (D1, D2), stopped proliferation earlier (at p5-6), while others, slower proliferating cultures of fetus-pathological gestation (D3, D4), took longer time to achieve senescence (at p7-8). Morphological changes of cells were observed during passaging, where AF-MSCs cultures from different donors presented a typical MSCs morphology with the appearance of a varied proportion of flattened cells (Figure 1(a)). The comparison of images of cell cultures during passaging showed enlarged and flattened morphology cells with increased cytoplasmic granularity and frequency for positive staining with senescence-associated *β*-galactosidase (SA-*β*-gal) at late passages (Figure 1(c)). These senescence-associated changes occurred later in AF-MSCs samples (D3, D4) that showed relatively lower level of proliferation.

**Figure 2:**
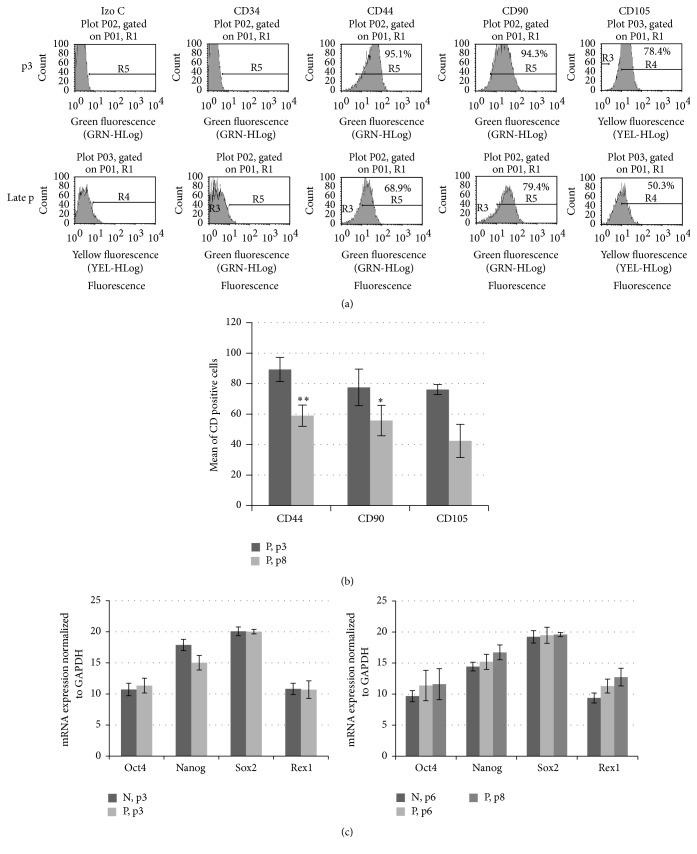
Immunophenotype and stemness markers of MSCs derived from AF of normal and pathological gestation during cultivation. (a) Representative flow cytometry histograms of cell surface antigen markers of MSCs obtained from AF of normal gestation (N) at passage 3 and the late passage 8. Cells were positive for staining with CD44, CD90, and CD105 antibodies and negative for CD34 antibody. The appropriate IgG isotype was used as a control. (b) The percentage mean of CD44, CD90, and CD105 positive cells in MSCs cultures derived from AF of pathological gestation (P) at passages 3 and 8. (c) The expression of* Nanog*,* Oct4*,* Sox2*, and* Rex1* from AF samples of normal (N) and pathological gestation (P) at passages 3, 6, and 8 was analyzed by RT-qPCR and normalized to GAPDH expression levels. Results are presented as the mean ± SD (*n* = 3, from each group). ^*∗*^ *P* ≤ 0.05 and ^*∗∗*^ *P* ≤ 0.01 were considered as significant changes.

Concomitantly, the analysis of flow cytometry was performed to determine immunophenotype representing one of the major parameters for the characterization of MSCs cultures. As shown in Figure 2(a), MSCs from AF samples of normal gestation at early passages were characteristic in their immunophenotype, showing over 90% of cells positive for CD44 and CD90 and over 75% for CD105 but were negative for the hematopoietic marker CD34. The expression of those markers decreased at late senescent passages (by about 27–15%, resp.). Fetus-pathological samples (Figure 2(b)) at passage 3 maintained quite similar but variable level of cell surface markers CD44 and CD105 with a lower extent of mesenchymal marker CD90. At senescent passage 8, the expression of all phenotypic markers decreased about 25%, while the expression of CD105, which is involved in cell proliferation, dropped by about 40%.

To confirm stem cell origin of AF-MSCs, we performed RT-qPCR analysis of the main transcription factors from AF samples of healthy donors (*n* = 3) and with fetal abnormalities (*n* = 3). Oct-4 and Sox2, involved in pluripotency or self-renewal, and Rex1, critically important in maintaining proliferative state in MSCs, were expressed at quite similar level in all AF samples at early and late passages (Figure 2(c)) while the expression of Nanog was relatively lower in fetus-pathological samples at passage 3. mRNA expression of stemness markers revealed alterations in the levels during passaging with variability in Oct4 expression in fetus-pathological samples. At late passage (p8), the increased expressional levels of Nanog and Rex1 were found in those samples as compared with passage 6, although the differences were not statistically significant.

### 3.2. Senescence-Associated Molecular Changes in MSCs Cultures

**Figure 3:**
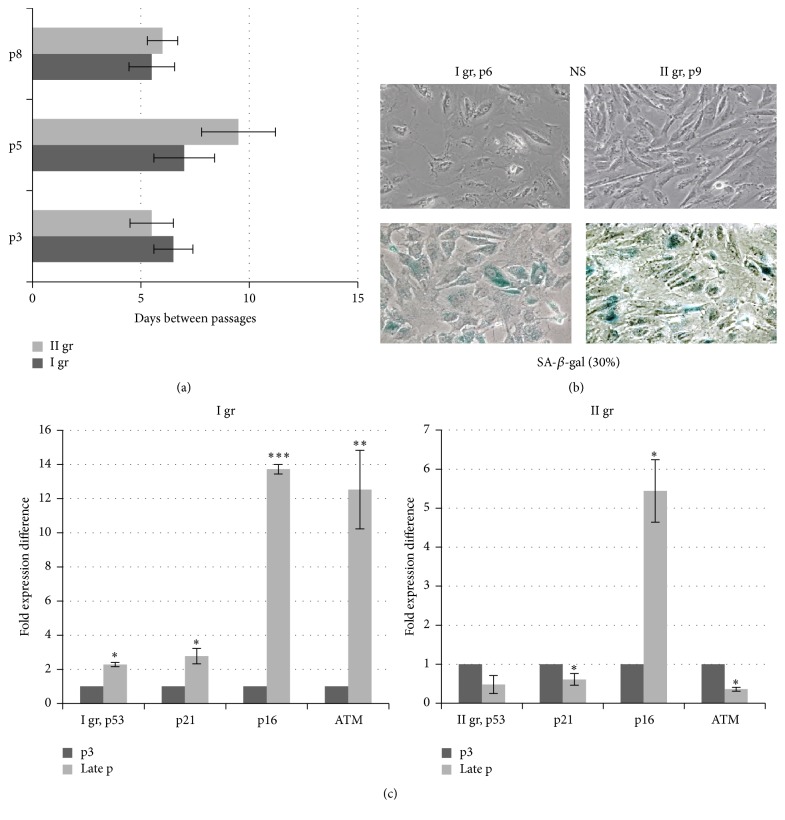
Characterization of senescence-associated markers in MSCs cultures during cultivation. (a) Differential proliferation of MSCs in two groups of cell cultures (I and II) during cultivation to passages 3, 5, and 8 (*n* = 3, for each group). (b) Nonstained (NS) and SA-*β*-gal positive cells at passages 6 and 9 in two groups of MSCs cultures. (c) Differential mRNA expression of senescence-associated markers in MSCs cultures (I gr and II gr) at passage 3 and the late passage was determined by RT-qPCR. Normalization to GAPDH and fold expression difference compared with passage 3 calculated using a comparative threshold cycle delta-delta Ct method. The data is presented as the mean ± SD (*n* = 3, from each group). ^*∗*^ *P* ≤ 0.05, ^*∗∗*^ *P* ≤ 0.01, and ^*∗∗∗*^ *P* ≤ 0.001 were considered as significant changes.

As is shown, during passaging of cell cultures derived from two AF sources, MSCs maintained their characteristic features such as immunophenotype and stemness factors, but the proliferative potential was seriously affected at late passages. Based on the observation that MSCs cultures differently undergo senescence in the course of cultivation, group I represents faster proliferating cell culture that earlier became senescent (at p5-6) and group II includes slower proliferating and undergoing senescence cultures (at p8-9) (Figure 3(a)). We observed that MSCs senescence was accompanied by typical morphological changes and cells became enlarged and flattened with enhanced SA-*β*-gal activity (Figure 3(b)). The same amount (about 30%) of SA-*β*-gal-positive cells was detected in groups I and II at p6 and p9 (denoted as late passage), respectively. To gain insight into the molecular characteristics of MSCs senescence, we analyzed changes in mRNA expression profiles of classical senescence-associated markers, p16INK4A (p16), p21WAF1 (p21), p53, and ATM (Ataxia telangiectasia mutated protein kinase), in both groups of cell cultures at early (p3) and senescent late passages paralleled with the monitoring of cellular morphology.

As demonstrated mRNA expression analysis determined RT-qPCR, senescent AF-MSCs cultures showed increased* p16* expression (Figure 3(c)) with more pronounced changes (about 14-fold versus p3) in group I as compared with group II (about 5.5-fold versus p3). The level of* p16* expression was inversely correlated with the proliferation capability. Surprisingly, we observed the increase in* p21* and* p53* expression in faster senescent cells from group I and the decrease in the level of both in slower senescent cultures from group II at late passages compared with early passage cells. The analysis of the expression of* ATM* that plays a role in cell cycle delay after DNA damage showed a positive correlation between increased expression levels of* ATM* and* p53/p21* in senescent cultures from group I and a negative one from group II. The data demonstrate that MSCs cultures differently underwent cycle arrest together with the decline of the efficiency of proliferation and the appearance of senescent morphology.

**Figure 4:**
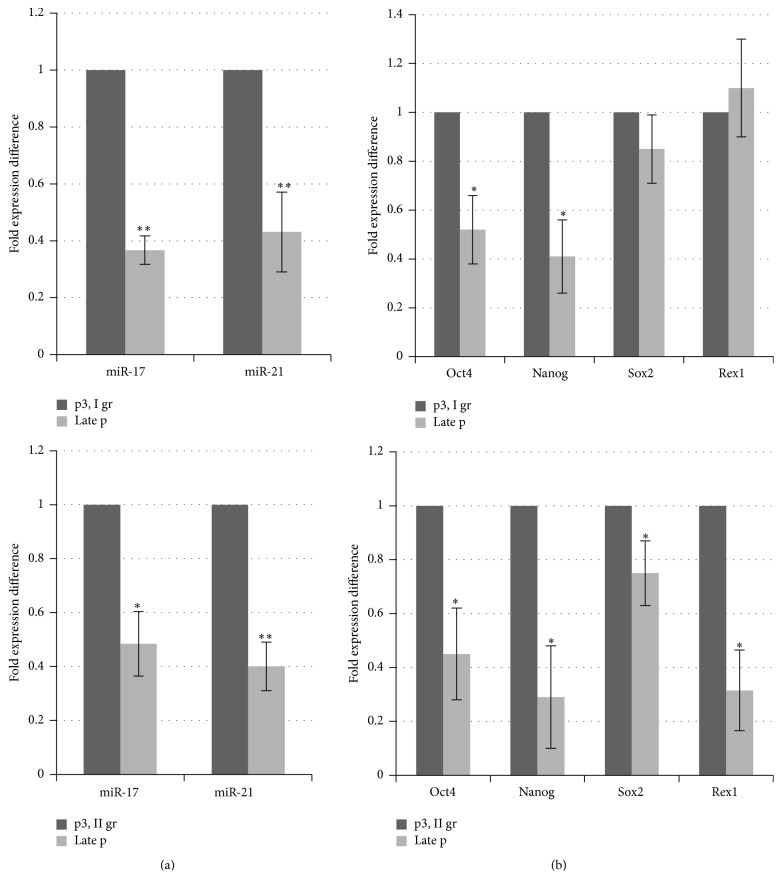
Differential expression of miRNAs and stem cell markers in MSCs cultures during passaging. RT-qPCR analysis of miR-17 and miR-21 (a) and Nanog, Oct4, Sox2, and Rex1 (b) from cell cultures of two groups (I and II) at passage 3 and the late passage. Normalization to calibrator sample (a) or GAPDH (b) and fold expression difference as compared with passage 3 calculated using a comparative threshold cycle delta-delta Ct method. The data is presented as the mean ± SD (*n* = 3, from each group). ^*∗*^ *P* ≤ 0.05 and ^*∗∗*^ *P* ≤ 0.01 were considered as significant changes.

Next, we focused on the expression analysis of miR-21 and miR-17 using RT-qPCR to evaluate the role of those miRNAs in the fate of MSCs during cultivation. The data presented in Figure 4(a) shows significant downregulation of both miR-17 and miR-21 in senescent cell cultures from group I and group II as well. Here we observed difference in fold changes of miR-17 expression ranging up to 2.7- and 2-fold downregulation in group I and group II, respectively, and quite similar decrease in miR-21 expression in samples of both groups (2.3–2.5-fold, resp.).

In order to evaluate a possible link between miRNAs and self-renewal regulating factors in senescent MSCs, we performed the comparative RT-qPCR analysis of essential transcription factors Oct4, Nanog, Sox2, and Rex1 in samples of MSCs cultures from group I and group II during culturing from early (p3) to late senescent passages. As shown in Figure 4(b), senescence of cells from group I caused a decrease in the expression of Oct4 and Nanog and nonsignificant changes in Sox2 and Rex1 levels. MSCs from group II showed a statistically significant decrease in expression levels of Oct4, Nanog, Sox2, and Rex1 at senescent passage compared with those at early passage (p3). Likewise, miR-17 and miR-21 may regulate the proliferation and senescence of MSCs through the effects on components of cell cycle machinery and possibly by the interaction with the transcription factors involved in cell proliferation and self-renewal.

### 3.3. Molecular and Epigenetic Alterations Associated with Senescence of MSCs from AF of Normal Gestation and with Fetal Abnormalities

**Figure 5:**
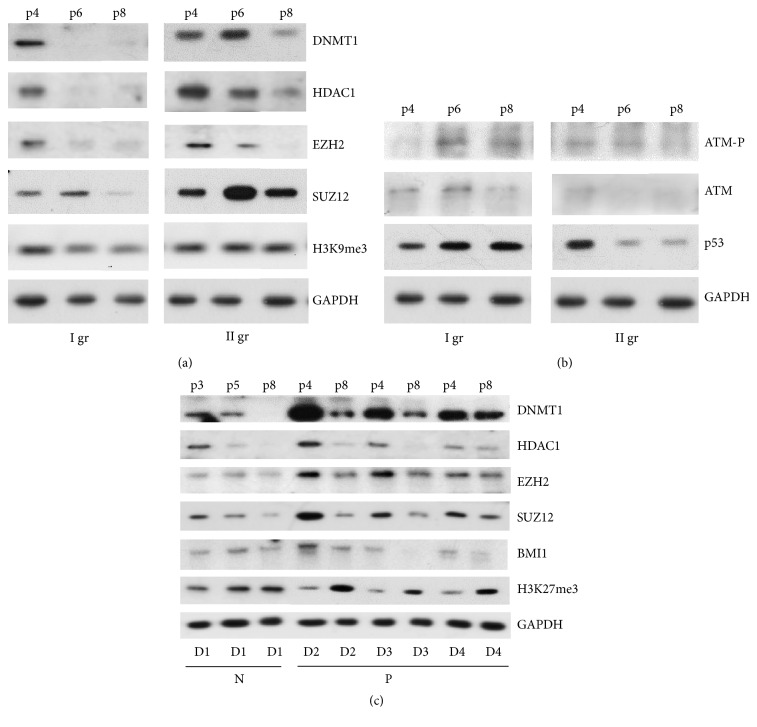
Epigenetic changes during passaging of MSCs cultures derived from AF of normal and pathological gestation. Lysates from cell samples of healthy (N) donors (D) and with fetal abnormalities (P) were subjected to Western blot analysis to monitor the expression of proteins using the indicated antibodies. (a, b) Representative blots of proteins from cell cultures of two groups (I and II) during culturing at passages 4, 6, and 8 and (c) from cell cultures derived from AF of normal gestation (D1) and individual donors (D2–D4) carrying fetus abnormalities. The data is representative of at least two gels showing similar results.

Next, we analyzed the functioning of the epigenetic regulatory factors HDAC and DNMT during the cellular senescence process. As shown in Figure 5(a), the level of DNMT1, which is constitutively expressed in proliferating cells for the maintenance of preexisting DNA methylation, decreased in MSCs cultures from groups I and II with the onset of cell senescence process and became almost undetectable at passage 6 (in group I) and at passage 8 (in group II). Additionally, the ATM analysis at protein level showed MSCs culture state before entering a growth arrest typical of senescence. In MSCs cultures of group I, phosphorylated ATM protein (ATM-P) accumulated at passages 6 and 8 concomitantly with the increase in the level of p53, while nonphosphorylated ATM was found at passages 4 and 6 with the apparent decrease at late passage (p8) (Figure 5(b)), showing cell cycle arrest after DNA damage-induced formation of double-strand breaks. In MSCs cultures II, less remarkable expression of ATM-P and ATM, gradually decreasing during passaging and in parallel with a marked decrease in p53 expression, was evident similarly as was demonstrated in Figure 3(c).

The analysis of HDAC1 and PRC2 polycomb group proteins, EZH2 and SUZ12, in samples of groups I and II demonstrated the expression profile similar to that of DNMT1 and HDAC1 (Figure 5(a)). In faster senescent cells from group I, a mark of constitutive heterochromatin, H3K9me3, showed decreased expression levels during passaging by a contrast to unchanged levels of this mark in cells from group II (Figure 5(a)). As shown in Figure 5(c), a decrease in the levels of DNMT1 and HDAC1 at late passages occurred in all cell cultures derived from individual AF samples from donors (D) of normal (N) and fetus-pathological (P) gestation and represented the extent of replicative ability of individual culture. For example, in sample from donor D4 (carrying Down's syndrome), where MSCs senescence process was delayed, DNMT1 and HDAC1 expression remained higher than in other samples at the same passage (p8). Similar expression changes in the levels of PRC2 proteins, EZH2 and SUZ12, and PRC1 component BMI1 were noticed in AF samples of the same donors, demonstrating their implication in MSCs senescence process (Figures 5(a) and 5(c)).

The inverse correlation between expression levels of histone H3K27me3 and EZH2, which specifically trimethylates histone H3 at lysine (K) 27, was found in senescent cells from fetus-affected AF samples, showing dynamical changes in chromatin structure during cell senescence. This mark accumulation occurred earlier and was maintained over passaging of faster senescent culture from sample D1. The results indicate that the epigenetic modifying enzymes, PRC2 and PRC1 complex proteins, and repressive histone modifications together with miRNAs cooperatively participate in senescence process of AF-MSCs cultures derived from healthy donors and individual donors with fetus malignancy.

## 4. Discussion

In our previous study, we demonstrated that AF-MSCs cultures from second and third trimester of normal pregnancy exhibited characteristic stem cell futures by the expression of specific cell surface markers and their ability of multipotential differentiation [28]. To date, little is known how AF-MSCs subpopulations derived from normal and fetus-affected donors change during culture expansion. As shown in this study, some MSCs cultures derived from AF of defected pregnancy during passaging displayed some variations in their phenotype (CD44+, CD90+, and CD105+) and proliferation potential. MSCs from different donors presented a typical elongated and spindle-shaped morphology at early passages to the appearance of various proportions of enlarged cells compatible with morphology of cells entering senescence. AF-MSCs samples showed interindividual variability in proliferation capability and time to reach senescence as was described in previous studies [29–31]. The data [24] support the notion of heterogeneity in MSCs cultures and also with regard to replicative senescence. There are evidences about exiting differences in the long-term proliferative capability of MSCs isolated from different donors and a negative correlation between donor age and MSCs proliferative capacity [13]. Additionally, proliferation rates in different AF samples highly varied from indefinitive exponential growth length to abruption by senescence without any explanation [13]. The observations about growth disadvantages due to fetus abnormalities are in line with several other studies, which indicated unusual growth failures related to fetal aneuploidy [32], aneuploid karyotypes, and gestation age as well [33] with lower growth rate in cultures of third trimester than those of first trimester [34].

Here we demonstrated that cultivation of different MSCs samples derived from AF of normal and pathological gestation leads to senescence and is related to morphological and molecular characteristics expressed differently in rather early passages. During cultivation of AF-MSC samples (from p3 to p8), the alterations in gene expression levels of senescence-associated markers (p16, p21, p53, and ATM) were correlated with the population proliferation and senescence abilities (according to the percentage of SA-*β*-gal-positive cells). In faster senescent MSCs cultures,* p21*,* p53*, and* ATM* expression notably increased but decreased in cultures with delayed senescence (Figure 3). However, both cultures contained elevated levels of senescence marker p16. Controversial data exists in senescent bone marrow MSCs regarding the expression of* p21*,* p53*, and* p16* from the upregulation of all of them [35] or of only* p16* [36] to a reduced expression of* p53* [37]. As known, cellular senescence is mainly regulated by p53/p21 and p16/pRb pathways, where the p53/p21 pathway mediates the replicative senescence and plays important roles in DNA damage response, while the p16/pRb pathway mediates stress-induced and premature senescence [38]. Based on our results, we suggest the involvement of both pathways in faster senescent MSCs, when p53 is induced and stabilized by phosphorylation of upstream kinases, including ATM and Chk2 [39–41]. Subsequently p53 upregulates transcription of p21, which activates Rb through the inhibition of cyclin E/Cdk2 complex, while activated Rb inhibits the transcription of E2F target genes, such as cyclin A and PCNA, causing a long-term cell cycle arrest [42]. Another Cdk inhibitor p16, which is mediated through transcriptional activation by Ets transcription factors, activates Rb through inhibition of cyclin D/Cdk4 and 6 complexes and accumulates in senescent cells causing cell growth retardation and arrest at G1 [21, 43, 44]. In a case of AF-MSCs from group II, where senescence process was delayed, only* p16* was upregulated, suggesting the involvement of p16/pRb pathway and supporting a notion that p16 and p21 play different roles in the initiation and maintenance of senescence cell cycle arrest [45].

It is not unexpected that DNMT controls cell senescence. The proliferative properties of MSCs are maintained by the expression of key pluripotency genes that downregulates cell cycle regulators, such as p16 and p21 [22], through a direct binding of Oct4 and Nanog to the promoter of DNMT1, which enhances its expression maintaining DNA methylation [23]. In our study, the reduction in the DNMT1 expression level during AF-MSCs senescence paralleled with downregulation of PRC2 complex proteins, EZH2 and SUZ12, and upregulation of* p16* and* p21* (Figures 3(c) and 5). The effective downregulation of EZH2 with greater effects in faster senescent AF-MSCs cultures (Figure 5) coincides with the notion that EZH2 is implicated in replicative senescence as well [46].

miRNAs are a class of small and noncoding RNAs of 18–25 nucleotides that generally degrade target gene expression at the posttranscriptional level [47]. Increasing evidence shows that miRNAs contribute to senescence-related changes in gene expression of many human cell types including MSCs [11, 48]. Recently, miR-17 (a member of miR-17-92 cluster) was demonstrated to be significantly downregulated in many aging model systems [48], suggesting its role as a novel biomarker of cellular aging. miR-17 has been shown to target genes involved in cell cycle control [49], including p21 [48, 50], through transcription activation by E2F and repression by p53, consequently activating the cyclinD1/Cdk4 complex [51, 52]. This is in agreement with our results, demonstrating a decrease in miR-17 expression (Figure 4), occurring together with increased levels of* p21* and* p53* at late passages in faster senescent cultures (Figure 3). In slower senescent cultures, miR-17 acts possibly through the regulation of E2F target genes involved in cell cycle control.

The role of miR-21 has been studied in many fields, including stem cell biology and aging [53]. In AF-MSCs, miR-21 has been shown to be expressed in high levels and is implicated in the regulation of proliferation potential and cell cycle arrest by direct targeting Sox2 and inhibiting its expression or reducing Oct4 and Nanog expression through an indirect mechanism [54]. Recent study [54] has shown that miR-21 induction in cycling, presenescence cells causes the decrease in the expression of Sox2, Nanog, and Oct4 and is associated with a decrease in cell proliferation rate and cell cycle arrest at G0/G1. In our study, miR-21 expression was significantly lowered in MSCs at senescent passages (p5–8) in coordination with decreased levels of Oct4, Nanog, and Sox2 (Figure 4). The suggestion that miR-21 is involved in promoting the senescence by targeting Sox2 is based on the data that Sox2 (but not Oct-4 and Nanog) was identified as a target of miR-21, being negatively regulated in spindle-shaped AF-MSCs [54]. In agreement with our data, the reduced expression of Oct-4 and Nanog with increased expression of p21 has previously been reported in MSCs at late passage. On the contrary, knock-down of p21 in late passages MSCs inhibited senescence and increased cell proliferation and expression of stemness markers [54]. Thus, downregulated expression of* Oct4* and* Nanog* was pointed as a potential candidate marker of the senescent state in stem cells [22, 23]. We also observed decreased expression of Rex1 in slower senescent MSCs cultures in association with a markedly decreased level of Nanog, which is transcriptional activator of Rex1, sustaining its expression.

The causal factors that mediate senescence process of AF-MSCs might include successive changes in the epigenetic state. Polycomb group proteins, which form multimeric complexes PRC1 and PRC2, have been shown to be implicated in replicative senescence [25–27]. The involvement of miRNAs and active/inactive histone marks at the promoter regions of cell cycle regulating p21 and p16 genes by targeting histone methyltransferases EZH1 is known. In turn, the inhibition of EZH1 in senescent MSCs leads to the demethylation of H3K27 and the activation of* p16* expression that is consistent with our results. In our study, we have demonstrated changes in repressive histone modifications, H3K9me3 (downregulated) and H3K27me3 (upregulated), during AF-MSCs culture expansion between passages 4 and 8. It is widely accepted that methylation of both marks is associated with gene silencing, but gene repression by H3K27me3 is related to facultative heterochromatin formation, whereas repression by H3K9me2/me3 is linked with constitutive (permanent) heterochromatin [55]. Methylation of H3K27 by EZH1 or EZH2 is associated with gene repression via the polycomb group proteins [46]. In proliferating cells, DNA is mostly found to be in a less condensed euchromatic state, allowing access by the transcription and DNA replication machinery. Conversely, senescent cells are characterized by the presence of densely packaged facultative heterochromatin, organized into structures named senescence-associated heterochromatin foci (SAHF) [56]. The data of recent study [24] indicate that DNA methylation changes during stem cells senescence are associated with repressive histone marks (including H3K9me3) rather than with bivalent modifications (H3K4me3 and H3K27me3). DNA methylation profiles revealed consistent senescence-induced hypermethylation in regions associated with H3K27me3 and H3K4me1/me3 marks, whereas hypomethylation is linked with chromatin containing H3K9me3. DNA hypermethylation was significantly enriched in genes that are either up- or downregulated at later passages [57]. A global loss of H3K9me3 and changes in heterochromatin architecture were detected in MSCs with futures of cell aging [58]. The causal role of HDAC1 in cellular senescence via changes in the structure of chromatin followed a decrease in HDAC1 levels and growth arrest was demonstrated also [59]. In this study we defined a significant reduction of HDAC1 linked with the extent of senescent process of AF-MSCs (Figure 5). Thus, within the context of chromatin, chromatin-modifying enzymes and repressive histone modifications are implicated in the defining of AF-MSCs fate via chromatin remodeling.

In conclusion, our study indicates that senescence process of MSCs during culture expansion and passaging includes alterations in the expression of cell cycle regulating genes, stemness transcription factors, miRNAs, and epigenetic modulators that have to be taken into account for AF-MSCs therapeutic application.

## Abbreviations

AF-MSCs: Amniotic-fluid-derived mesenchymal stem cells
BMI1: B lymphoma Mo-MLV insertion region 1
EZH1 and 2: Enhancer of Zeste histone H3K27 methyltransferase
GAPDH: Glyceraldehyde-3-phosphate dehydrogenase
Oct-4: Octamer-binding transcription factor 4
PRC: Polycomb repressive complex
RT-qPCR: Quantitative real-time polymerase chain reaction
Sox-2: Sex determining region Y-box 2
Suz: Suppressor of Zeste.
